# Perceptions of exercise and exercise instruction in patients with type 2 diabetes mellitus and sarcopenia : a qualitative study

**DOI:** 10.1186/s12877-022-03519-0

**Published:** 2022-11-22

**Authors:** Shangjie Che, Min Meng, Ya Jiang, Xiang Ye, Cuihua Xie

**Affiliations:** 1 Department of Endocrinology and Metabolism, Nanfang Hospital, Southern Medical University, No.1838, Guangzhou Avenue North, Baiyun District, Guangzhou, China; 2 School of Nursing, Southern Medical University, Guangzhou, China

**Keywords:** Type 2 diabetes mellitus, Sarcopenia, Exercise, Qualitative study, Knowledge-attitudes-practices

## Abstract

**Background:**

Exercise plays a major role in helping patients with type 2 diabetes mellitus and sarcopenia to increase muscle mass and muscle strength. However, little is known about perceptions of exercise and exercise instruction in these patients. This study aimed to explore the perceptions of exercise and exercise instruction from the patient’s perspective.

**Methods:**

In a descriptive qualitative study, semi-structured face-to-face in-depth interviews were conducted with 16 patients with type 2 diabetes mellitus and sarcopenia at a tertiary hospital. The Consolidated Criteria for Reporting Qualitative Research (COREQ) guidelines were followed to ensure rigor in the study. The interviews were analysed using a thematic analysis method.

**Results:**

Four themes and 13 sub-themes were identified in this study. The four themes were knowledge-attitudes-practices surrounding exercise, motivators and barriers regarding exercise, and attitudes towards professional exercise instruction.

**Conclusion:**

This study provides a detailed understanding of the knowledge-attitudes-practices, motivators and barriers regarding exercise among patients with type 2 diabetes mellitus and sarcopenia, as well as attitudes related to exercise instruction. The current findings can guide healthcare professionals, patients’ families, and policymakers to motivate patients to be physically active through policy initiatives and other types of incentives and programmes, such as providing more health education and holistic support, increasing family and friends’ companionship and care, and providing suitable exercise conditions.

**Supplementary Information:**

The online version contains supplementary material available at 10.1186/s12877-022-03519-0.

## Background

Approximately 90% of diabetes mellitus (DM) patients have type 2 diabetes mellitus (T2DM), data from the International Diabetes Federation (IDF) indicated that the global prevalence of DM reached 9.3% in 2019 and is expected to reach 10.9% (700 million) by 2045 [1]. This is a global public health problem and involves a substantial economic burden. Sarcopenia was recently identified as a complication of T2DM [2], which is a syndrome of progressive skeletal muscle loss associated with ageing [3], and these two diseases affect each other. Sarcopenia can contribute to the development and progression of T2DM, and insulin resistance, inflammation, advanced glycation end products (AGEs) accumulation, increased oxidative stress and vascular complications due to hyperglycaemia can all impair muscle and muscle strength [4]. A recent meta-analysis indicated that the prevalence of sarcopenia ranged from 8% to 36% in individuals < 60 years of age and from 10% to 27% in individuals ≥ 60 years of age [5]. The prevalence of sarcopenia in patients with T2DM was 18% [6], and the risk of sarcopenia in T2DM patients was 1.5 times higher than that in euglycaemic patients (odds ratio: 1.55; 95% confidence interval: 1.25–1.91) [7]. Patients with T2DM and sarcopenia have more impairment of muscle than the general population. Adverse outcomes associated with sarcopenia in DM patients include falls risk, depression, cognitive dysfunction, hypoglycaemia and the risk of death due to cardiovascular and non-cardiovascular disease [8]. Sarcopenia and frailty have emerged as third category of complications leading to considerable disability in addition to the traditional microvascular and macrovascular disease [9]. Therefore, the prevention and treatment of T2DM combined with sarcopenia is becoming an increasingly important issue in diabetes research.

Exercise is an effective, sustainable, and low-cost strategy for the treatment and management of DM that enhances glycaemic control, insulin signalling and lipids, as well as reducing low-grade inflammation, improving vascular function and reducing body weight [10]. Exercise is also considered to be a cornerstone of the prevention and treatment of sarcopenia [11], reducing the loss of muscle mass and strength and improving physical function and quality of life [12]. Nevertheless, less than one third of people with DM performed the level of physical activity recommended by the American Diabetes Association [13]. Physical inactivity is one of the major modifiable risk factors for global mortality, with an estimated 20%–30% increased risk of death among inactive individuals compared with those who exercise [14].

In recent years, several quantitative studies exploring the effects of aerobic and resistance exercise on patients with T2DM have proved that exercise is always beneficial to patients [15–17], relatively, qualitative studies have showed factors affecting patients’ exercise. For example, high levels of social interaction helped maintain activity levels in overweight and obese T2DM patients [18]. In addition to barriers such as lack of time, inability to link exercise to glycaemic control, inadequate physician attention, and physical limitations, the failure of healthcare professionals to follow relevant guidelines when giving exercise advice to patients remained a significant problem [19]. Moreover, it was appropriate to construct individualised exercise models on the basis of the exercise experiences of older Taiwanese patients with DM and sarcopenia, and necessary emotional support could be effective for preventing DM and sarcopenia [20]. However, our study assumes that, in addition to the experience of exercise, the exploration of perceptions and attitudes towards exercise in Chinese patients with these two diseases may also play a key role in improving individualised exercise instruction programmes. Therefore, this study was conducted to explore the following issues to help healthcare professionals develop appropriate exercise models for their patients: (1) the perceptions, attitudes, exercise routines and reasons for exercise in Chinese patients with T2DM and sarcopenia; (2) the motivators and barriers to exercise in these patients and their expectations regarding professional exercise instruction.

## Methods

### Study design

This qualitative study used a descriptive phenomenological approach involving a series of personal semi-structured interviews designed to provide an in-depth description of the phenomena associated with the perceptions of patients with T2DM and sarcopenia in relation to exercise. We also aimed to identify any motivators and barriers related to exercise in people with T2DM and sarcopenia. The study was conducted following the COREQ [21]. This descriptive phenomenological study was conducted from February 2022 to April 2022 at the Department of Endocrinology and Metabolism of a tertiary care hospital in Guangzhou, China.

### Recruitment and participants

Purposive sampling with maximum variation was adopted in this study. Inclusion criteria for patients with T2DM and sarcopenia were as follows: (1) aged 18 years and above, diagnosed with T2DM; (2) ability to communicate in Mandarin; (3) diagnosis of sarcopenia by walking speed < 0.8 m/sec and grip strength < 26 kg for men and < 16 kg for women [22]; (4) scoring > 4 on the SARC-F questionnaire as recommended by European Working Group on Sarcopenia in Older People (EWGSOP2) [23]. The SARC-F is a simple screening tool used to obtain self-reports from patients with signs of sarcopenia, comprising five assessment items: strength, walking assistance, getting up from a chair, climbing stairs, and falls; (5) voluntary participation. Patients were excluded if they had cognitive impairment or were unable to independently verbally communicate in Mandarin. Patients with T2DM and sarcopenia in our unit were inpatients receiving treatment. Ethical approval for this study was obtained from the ethical committees of Nanfang Hospital of Southern Medical University (NFEC-2022-093), and written informed consent was provided by all patients.

### Data collection

Patients who met the inclusion criteria were invited to start the interview when they felt well during their hospitalisation. Patients were invited to participate in semi-structured interviews. Thus, 16 face-to-face, semi-structured interviews were conducted in a separate examination room in the department, each lasting approximately 15–35 min, depending on the patient’s physical condition and the adequacy of the description of their perceptions. Patients were able to interrupt the interview if they wished. Patients were briefed on the purpose, format and approximate time of the interview prior to the formal interview. Demographic information and informed consent were obtained within 10–15 min prior to the interview, and the interview was recorded with the consent of the patient. Patients were interviewed in accordance with the following interview guidelines (Table 1). The interview guide used in this study was prepared on the basis of literature reviews and clinical observations, then agreed by consensus among the study team. To minimise bias, a trained female researcher, who was not part of the endocrinology staff, conducted the semi-structured interviews. A trusting relationship was established between the researcher and the patients. To increase patients’ confidence and ensure that patients were free to talk about their perceptions, the researcher used appropriate silences, endorsements and follow-up questions, and remained neutral, non-judgemental and unbiased during the interview. The interview guide was only used as a reminder to prevent the researcher from missing important content. The researcher made adjustments to the order, content and questioning style of the interview as appropriate on the basis of the individual responses, and recorded the feelings, emotions and non-verbal expressions of the patients during the process. Long and short questions were asked alternately to maintain an appropriate pace of the interview. In the recorded data, patients were given code names rather than their real names. Data collection and data analysis were carried out simultaneously. After interviewing 14 patients, no new themes emerged in the last two interviews, indicating that data saturation had been reached, and we stopped the interviews.

**Table 1:** Interview guidelines

1. Please tell me how you understand diabetes and sarcopenia?
2. Please explain your experiences and feelings about exercise ?
3. Please describe your experiences regarding the barriers and facilitators that influence exercise? What efforts have you made to keep exercising?
4. Please tell me what kind of support you have received regarding exercise?
5. Please tell me what kind of support you need to perform exercise?
6. Please tell me about your opinions of and attitudes towards the professional instruction of exercise?

### Data analysis

Within 24 h of each interview, the recorded data were transcribed verbatim by the interviewer and the transcripts were reviewed to check their accuracy. The data were analysed using thematic analysis [24], and no data analysis software was used. The transcripts were read repeatedly by both researchers to familiarise themselves with the transcripts, and data relating to the initial codes were then extracted from the entire set of textual data. Candidate themes were identified in the data by iteratively comparing and integrating individual codes and reporting models. We reviewed themes on the basis of their consistency with the coded text and the entire dataset, and the research team reviewed the ongoing coding together for validation, identification of irrelevant or incorrect codes, and discussion of emerging patterns. This process provided the research team with an opportunity for ongoing observation and reflexivity. We refined and named the themes. Finally, quotations were selected for use in the results section. Participants did not provide feedback on the findings. Therefore, to ensure credibility, categorisation was discussed within the research team until consensus was reached. In addition, the methods and results (including citations) were described in detail on the basis of the recommendations of the COREQ. All authors were female, professionally trained health professionals with a nursing background. Raw data (audio recordings, transcripts, field notes), coding patterns, coding transcripts, and thematic reports were archived by date to provide an audit trail.

## Results

### Demographics

This study reached data saturation with 16 patients with T2DM and sarcopenia. Patient demographics are shown in Table 2.

**Table 2:** Demographic characteristics of interviewees

Variable	Frequency (%)
Patients’ characteristics (P = 16)	
**Age (years)**	
≤ 50	1 (6.3)
51~60	1 (6.3)
61~70	6 (37.5)
71~80	5 (31.3)
81~90	3 (18.8)
**Gender**	
Men	7 (43.8)
Women	9 (56.3)
**Occupational status**	
Employed	1 (6.3)
Unemployed	2 (12.5)
Retired	13 (81.3)
**Education**	
Junior diploma or below	10 (62.5)
Above Junior diploma	6 (37.5)
**Duration of diabetes**	
10 ~ 19	11(68.8)
20 ~ 29	5(31.3)
**Family monthly income (CNY** ^**a**^ **)**	
3000–5000	6 (37.5)
5000–8000	10 (62.5)

^a^ CNY is Chinese Yuan

### Themes

Four themes were identified regarding the experiences of patients with T2DM and sarcopenia: theme (1) knowledge-attitudes-practices regarding exercise; theme (2) motivators for exercise; theme (3) barriers to exercise; and theme (4) attitudes towards professional exercise instruction. Themes and sub-themes are shown in Table 3, and exemplar quotes from patients are shown in Supplementary Table 1.

**Table 3:** Study themes and sub-themes

Themes	Sub-themes
Knowledge-Attitudes-Practices	Knowledge about exercise
	Attitudes towards exercise
	Practices of exercise
Motivators	Desire for health
	Positive feelings regarding exercise
	Social support
Barriers	Physical discomfort
	Psychological factors
	Poor exercise conditions (weather, exercise area, assistive devices, time)
Attitudes towards professional exercise instruction	Urgent need for exercise instruction
	Fear of intensity of the instructed exercise
	Financial constraints

#### Theme 1 knowledge-attitudes-practices regarding exercise

1. *Knowledge about exercise* Patients (4/16) briefly stated that exercise was beneficial for DM, and expressed an understanding of the effect of exercise on improving blood glucose. Nonetheless, some patients believed that, for patients with T2DM, exercise should be strictly controlled in terms of intensity and frequency, and that over-exercise could lead to adverse consequences. Generally, the information they had was scattered and non-authoritative.

*N4 (male, 73 years of age): I got some diabetes information from doctors, books and diabetic patients suggesting that diabetic patients shouldn’t do too much exercise.*

*N16 (male, 50 years of age): I heard that “exercise benefits people with diabetes” from doctors, and that we need to do more exercise, like…walking is a good choice.*

2. *Attitudes towards exercise* Some patients (3/16) said they believed and accepted that “exercise helps treat DM” and were willing to do a sufficient amount of exercise.

*N12 (female, 85 years of age): I consider that exercise helps treat diabetes, and I believe it.*

*N15(male, 83 years of age): I keep doing exercise for as long as I can. I know it’s really good for my body.*

3. *Practices of exercise* Although patients knew the benefits of exercise and had positive attitudes towards exercise, not all of them (4/16) performed exercise in their lives, with only some patients engaging in different types of exercise regularly. Exercise mentioned by patients included walking, hiking, and morning exercise in the park, with walking being the most popular exercise.

*N13 (female, 90 years of age): My body feels weak now, and all I can do is walk around at home following the walls.*

*N4 (male, 73 years of age): I used to do morning exercise in the park several months ago, but I haven’t been doing any exercise recently.*

#### Theme 2 motivators regarding exercise

Motivators (desire for health, positive feelings associated with exercise, and social support) were identified based on statements by patients. Whether these factors were considered motivators or barriers was dependent on the individual’s unique situation.

1. *Desire for health* Some patients (5/16) expressed a desire for good health and early recovery from illness, believing that they had to take actions and exercise to control their blood glucose, relieve pain, and improve physical function. Patients were also very concerned that a sedentary lifestyle or inactivity would result in reduced physical function and worsen the symptoms of their disease, such as stiffness, numbness, and weakness in their limbs.

*N1 (female, 76 years of age): I keep going for walks even though I’m in hospital, because my feet feel weak, painful, and stiff if I don’t take a walk.*

*N14 (female, 71 years of age): Yes, the more you lead a sedentary lifestyle, the more trouble your body will run into.*

2. *Positive feelings regarding exercise* Patients (3/16) described their positive feelings regarding exercise, reporting that they felt happier, more comfortable, and energetic after exercise, as well as improving their self-care ability.

*N14 (female, 71 years of age): Doing exercise regularly makes me feel more energetic and increases my ability to take care of myself.*

3. *Social support* Patients (4/16) highlighted the importance of social support, especially care and advice from family members, and companionship and encouragement from friends. Support was one of the key factors that motivated patients to keep exercising. Patients saw their exercise partners as an essential presence in exercise, which made the exercise more fun and interesting.

*N11 (female, 76 years of age): It’s really interesting to do exercise with friends. I can chat with them during exercise, so I don’t even feel tired and the time passes quickly. I quite enjoy it.*

*N3 (male, 66 years of age): I’m quite willing to do exercise when my children support and encourage me, because they give me energy and courage.*

#### Theme 3 barriers to exercise

1. *Physical discomfort* Patients (4/16) reported that one of the most important factors that prevented them from exercising was physical discomfort, which included weakness in the arms and legs, fatigue, and physical pain. Patients reported that it was inconvenient for them to move, and sometimes even standing was difficult.

*N8 (male, 60 years of age): My feet feel very weak and my lower back feels painful because of my lumbar intervertebral disc, so it’s difficult for me to stand up, let alone exercise.*

2. *Psychological factors* Patients (3/16) described that when they were in a negative mood, their enthusiasm and motivation to engage in activities decreased, which made it difficult for them to exercise.

*N5 (female, 77 years of age): Sometimes I feel unhappy and I just want to lie in bed alone, which means that I don’t have the energy or enthusiasm to do exercise.*

3. *Poor exercise conditions (weather, exercise area, assistive devices, time)* Patients (4/16) mentioned that a lack of suitable weather and exercise places had the greatest impact on their willingness to do outdoor exercise, especially among older people. The lack of assistive devices (such as crutches or walkers) also deterred patients who were more frail from exercising, as they need equipment to support their bodies. In addition, some female patients reported that they were too busy with daily chores, farm work and childcare, so they did not have the extra time and energy to engage in additional physical activity. The poor exercise conditions affected some patients’ willingness to exercise.

*N15 (male, 83 years of age): I insist on going for a walk every day in good weather, but if it’s raining I don’t go out because……I’m too old and I am…afraid of falling, which would be terrible and dangerous.*

*N2 (female, 63 years of age): I feel very busy and tired with all the grocery shopping, cooking, laundry, and looking after my grandchildren, and I have absolutely no time or energy to exercise.*

#### Theme 4 attitudes towards professional exercise instruction

1. *Urgent need for exercise instruction* Patients (5/16) expressed an urgent need for exercise guidance from healthcare professionals. However, most of the information patients received in hospital was basic advice from healthcare professionals (e.g., “More exercise is good for maintaining blood glucose”) but no specific type, frequency or intensity of exercise was given. Each patient chose to exercise according to their own habits or preferences.

*N9 (female, 68 years of age): I definitely need healthcare professionals’ guidance about exercise, I really need that, but every time I feel like the doctors and nurses just tell me to exercise more, which is too vague.*

2. *Fear of intensity of the exercise instructions* Patients (3/16) mentioned that they were unsure whether the intensity of the exercise instruction from healthcare professionals was suitable for them, that they were afraid that excessive intensity would cause physical harm or that they would feel frustrated if they could not achieve their intended exercise plans. Additionally, patients expressed a wish that professional exercise instructions were based on their own specific physical characteristics or limitations.

*N12 (female, 85 years of age): I wouldn’t accept professional guidance unless I’m able to do it. I wonder whether I could do it or not, because I would feel frustrated and useless if I couldn’t achieve the exercise goals.*

3. *Financial constraints* Some patients (3/16) were concerned about whether they would need to pay for exercise instruction. Patients expressed that if exercise instruction was not free they would give it up to avoid increasing their economic burden, which was already heavy after spending a substantial amount of money on hospital bills related to their DM.

*N10 (male, 68 years of age): It would be better if the exercise instruction was free. That would be the most helpful and important thing for me. We don’t have much money and my medicine is expensive, so life is really hard. We are afraid to get sick because it’s really expensive.*

## Discussion

The current qualitative study provides insight into the perceptions of exercise among patients with T2DM and sarcopenia, and our findings provide a holistic view of the multifaceted issues surrounding exercise in these patients. Patients’ knowledge-attitudes-practices, their motivators and barriers to exercise, and their attitudes regarding exercise instruction were found to be four important aspects of the implementation of exercise intervention.

With the global increase in the prevalence of DM, assessment of DM knowledge, attitudes and practices is considered to be important for guiding behavioural change in people with DM [25]. This is also the case among patients with T2DM and sarcopenia. Knowledge is a prerequisite for health behaviour change [26]. Similarly, our findings suggested that the patient’s deficiency in disease and exercise knowledge was one of the reasons that made it difficult for them to start or stick to exercise, because they were unaware of the relationship between exercise and disease and were not sure about how to do suitable exercise. Also, we found that misconceptions about DM were common among older, less educated, and lower-income DM patients, which is consistent with the findings of a previous study [27]. Besides, we found that the sources of patients’ knowledge were varied and included not only guidance from healthcare professionals but also from social media and television, friends and family members, books and newspapers. Additionally, people’s use of social media increased significantly during the Corona-virus disease 2019 pandemic. However, experts’ advice was the most positive source of information [28], and although social media can be used to disseminate health improvement measures and promote the adoption of healthier behaviour patterns [29], information on social media is sometimes misleading [28]. Limited health information and/or online literacy may lead to misunderstanding when assessing medical data on the web [30], which can influence individuals to make harmful or counterproductive behavioural changes. Therefore, promoting scientific guidance from healthcare professionals on patient’s exercise will be essential in the future, as well as educating patients about the accuracy of information from different unofficial sources to improve their e-health literacy.

Beliefs reflected patients’ strongly felt perceptions of the alternatives available to reduce the threat of disease. Risk perceptions and positive outcome expectations may act as triggers for participation in physical activity. The current findings indicated that one of the reasons patients held positive beliefs regarding exercise was that they personally felt a significant improvement in their blood glucose, physical strength and energy after adhering to exercise, which made them more convinced of the advantages of exercise and more willing to maintain an exercise habit, leading to a virtuous circle. Beliefs about physical activity are an underlying factor in physical activity behaviour [31].

According to our results, some patients adhered to physical activity in their lives, which is consistent with the results in a cross-sectional study of patients with DM alone [13]. Despite patients showing a good level of knowledge and positive attitudes in previous studies, their practice outcomes remained poor [32, 33]. The most popular exercise programme in this study was walking, and the most popular exercise for people with only DM was found to be walking in Vanden’s study [34]. However, patients who adhered to the exercise programme did not perform resistance or endurance training, and the effectiveness of resistance training in strengthening muscles in older patients with only T2DM has been demonstrated previously [35, 36]. Positive effects of training on improving muscle strength and physical function in patients with sarcopenia alone was reported in a previous study [37]. Hence, future exercise design for people with T2DM and sarcopenia should be individualized, and resistance training should be considered to help them perform a sufficient amount of muscle training exercise. Clinical staff should also focus on what patients can do rather than what they cannot do, advocating for higher exercise levels along the range of mobility, with “inactivity” at one end and “recommended daily exercise levels” at the other [38]. Patients’ confidence in exercise should be enhanced by encouraging a gradual progression.

Patients with T2DM and sarcopenia in this study identified motivators and barriers to exercise. Patients expressed a strong desire to be healthy, which was a significant facilitating factor. Patients’ concerns about the negative health outcomes of being sedentary or inactive prompted them to do simple exercises to maintain physical function even though they were reluctant to exercise, representing an internal struggle. Lin et al. proposed that healthcare professionals should pay more attention to occasional internal struggles regarding resistance to exercise among patients [20], and it is important to increase patients’ enjoyment and motivation to exercise. Additionally, as mentioned earlier, positive feelings associated with exercise were also a central factor in patients’ adherence to exercise, and this feeling of perceived benefit from exercise enhanced patients’ positive beliefs regarding exercise. The current results revealed that social support from family and friends played an important role in patients’ exercise adherence, which is consistent with Schmidt’s study of patients with only T2DM [39]. Encouragement and support from family members may give patients confidence and motivation, and a lack of family concern can be a deterrent to exercise.

Some patients highlighted the physical limitations that greatly hindered their ability to exercise, reporting that they often felt weak and tired in their arms and legs because they were ill, and they also exhibited reduced mobility and poor physical endurance, which is consistent with the symptoms of sarcopenia [40]. Patients reported that the physical discomfort caused by diseases made it difficult for them to move around. In these situations, even if the patient wanted to walk or do other exercise, they were unable to do so. Therefore, more consideration should be paid to older patients who experience physical discomfort, and those who are unable to exercise regularly. Some patients experience psychological suffering from mobility problems, and future research should focus on how to actively engage this group in individualised, evidence-based, and effective exercise programmes that suit them. Negative emotions can also affect engagement in exercise, even among patients who routinely exercise but do not feel like exercising when they are in a bad mood, or are sometimes overwhelmed by frustration or sadness. This finding is in accord with those of a previous study, which found that emotional support was a motivator for exercise in patients with T2DM alone [34]. Healthcare professionals should closely assess patients’ psychological state and provide timely psychological care and intervention to encourage patients to maintain a positive state of mind.

In addition to physical and psychological factors that can affect patients’ exercise, poor exercise conditions can also hinder their exercise, such as weather, exercise areas and assistive devices. Weather is a major challenge for outdoor sports, which is consistent with the findings of Lin’s study [20]. Poor weather conditions (e.g. too cold, too hot, or too rainy) can significantly delay people’s participation in outdoor activities and further affect their willingness to exercise [41]. Moreover, participation in outdoor sports in adverse weather conditions increases the risk of accidents, particularly among older people population [42], most of whom have limited mobility and cannot easily leave their homes. Therefore, more indoor activities should be actively pursued, especially in the context of the coronavirus disease 2019 pandemic, during which the variety and enjoyment of indoor activities has been widely researched, because such activities are more convenient and accessible than outdoor activities. A lack of necessary exercise areas and facilities can also significantly reduce people’s interest and habits regarding exercise, suggesting that governments and communities should take steps to match community residents with appropriately sized open areas and adequate exercise facilities, and to provide appropriate conditions for people to exercise outdoors as much as possible. Healthcare professionals can group people of the same age in close geographical proximity and encourage them to exercise together during the week to increase their motivation and adherence to exercise [43]. Assistive devices such as crutches and walkers provide necessary support for patients with reduced mobility, and families and healthcare professionals should take this into account. Another barrier to exercise reported by our participants was a lack of time, which was particularly pronounced in female patients. Women are often caregivers and bearers of household chores in the traditional Chinese cultural context, which causes them more physical and mental stress and increases the risk of negative health outcomes. Therefore, it is important to encourage male partners’ assistance with domestic work and promote access to labour-saving devices to simplify various tasks and reduce the time spent performing domestic activities. These steps have been shown to have positive implications for women’s health [44], resulting in more free time to engage in exercise or other activities with health benefits.

In the current study, patients reported a strong need for professional exercise advice and guidance, and mentioned that in clinical settings healthcare professionals rarely gave them specific exercise guidance that was based on scientific knowledge. Some patients in our sample were keen to exercise in a way that would benefit their bodies, but they did not know what type of exercise was most appropriate for their bodies and health conditions, and they worried that performing exercise casually without professional guidance may cause physical harm. Advika emphasised the importance of the roles of healthcare professionals, and that patient education, empowerment and counselling by healthcare professionals may contribute to adherence [19]. Doctors should not only encourage patients to exercise, but also provide them with standard advice on the type and duration of exercise. It is essential that doctors reiterate the need for exercise and the role it can play in improving patients’ health during each visit. Positive personalised exercise instruction helps to build a good doctor-patient relationship and may change the patients’ perceptions of exercise, thus improving compliance. Hospitals should organise uniform training for healthcare professionals in professional exercise instruction so that they can learn more about the medical expertise regarding exercise for patients in their unit. Vanden found that nurses play a direct role in instructing patients with only T2DM in physical activity, and that their instructions should include information about the safety of participation, the intensity and duration of exercise needed to achieve maximum benefits, and ways to address barriers to help patients to safely obtain adequate muscle exercise and increase their confidence, motivation, and compliance with exercise [34]. Moreover, patients in the current study expressed worries about financial constraints and the cost of exercise advice from healthcare professionals, which could increase the heavy financial burden they already face because of their T2DM and become a barrier for them. Adu found that financial constraints were also a barrier to self-management in patients with DM [45]. Regarding the availability and accessibility of healthcare resources, the government needs to provide financial support to patients to reduce their financial burden. For example, the cost of treatment for DM and sarcopenia could be covered by health insurance. In summary, healthcare professionals should develop individualised and appropriate exercise programmes by identifying barriers, motivators, and patients’ perceptions of professional exercise instruction to help increase patients’ motivation and participation in exercise.

## Strengths and limitations

The present study had several strengths that should be noted. This study was reported according to the COREQ guidelines (Supplementary File 1). To enhance the reliability of the study, any disagreements in design, methodology, data analysis and results were discussed by the research team until agreement was reached. Moreover, all data were analysed in a team-based manner, with the research team accurately converting the recorded data into transcripts during the data analysis process. To ensure the authenticity of the study findings, all themes were supported by patients’ quotations. To facilitate the transferability of the study, clear descriptions of the sampling method, inclusion criteria, exclusion criteria and patient characteristics were provided. During the study, the interview guide, recorded data, original transcriptions, records of the parsing transcription process, and study results were retained by the researchers.

Nonetheless, the current study involved limitations. Patients were recruited from a tertiary care hospital. Thus, the sample was not representative of all people with T2DM and sarcopenia, and recruiting patients in one city may limit the generalisability of the current findings to other areas and ethnicities. Therefore, the findings and conclusions should be interpreted with caution in terms of generalisation. Future studies should explore the exercise experiences of these kinds of patients in various types of hospitals and institutions and across ethnicities. Additionally, further studies exploring the perceptions of healthcare professionals are recommended to gain a comprehensive understanding of the topic.

## Conclusion

In conclusion, exercise is a simple and effective intervention helping to stabilize blood glucose and improve muscle strength. Our findings could provide evidence on how to motivate these patients to exercise more. More attention and comprehensive support are needed from healthcare professionals to give patients with T2DM and sarcopenia, helping them develop individualized exercise programmes that address barriers to exercise and strengthen the motivators to increase compliance with exercise and maintain physical function.

## Electronic supplementary material

Below is the link to the electronic supplementary material.

Supplementary Material 1. Consolidated Criteria for Reporting Qualitative Research (COREQ). 

Supplementary Material 2. Supplementary Table 1. Quotes from patients.

## Data Availability

The datasets generated and/or analysed during the current study are not publicly available due to privacy concerns but are available from the corresponding author upon reasonable request.

## Abbreviations

**T2DM**: Type 2 Diabetes Mellitus
**COREQ**: Consolidated Criteria for Reporting Qualitative Research
**EWGSOP2**: European Working Group on Sarcopenia in Older People
**DM**: Diabetes Mellitus
**IDF**: International Diabetes Federation
**KAP**: Knowledge, Attitudes and Practices
**AGEs**: Advanced Glycation End products
