# Supplementary material for: Perceptions of exercise and exercise instruction in patients with type 2 diabetes mellitus and sarcopenia : a qualitative study
Source: BMC Geriatr. 2022 Nov 22;22:892. doi: 10.1186/s12877-022-03519-0 (PMC9682829; doi:10.1186/s12877-022-03519-0)
Supplement: Supplementary file 2 — Supplementary Material 2. Supplementary Table 1. Quotes from patients. [file 12877_2022_3519_MOESM2_ESM.docx]

| **Supplementary Table 1**. Quotes from patients | | |
| --- | --- | --- |
| Themes | Subthemes | Quotes |
| Knowledge-Attitudes-Practices | Knowledge about exercise | N4 (male, 73 years of age): I got some diabetes information from doctors, books and diabetic patients suggesting that diabetic patients shouldn’t do too much exercise.  N15(male, age 83): I know that moderate exercise is good for people like me, but I was thinking that when I had diabetes, I had to control my diet and eat less than before, so where would I find the energy to go for a walk or do exercise? |
|  |  | N16 (male, 50 years of age): I heard that “exercise benefits people with diabetes” from doctors, and that we need to do more exercise, like...walking is a good choice. |
|  |  | N6(female, age 67)：You mean diabetes? Em……em,they said……actually I know nothing about it, but my husband said dancing could relieve your pain of arms, legs and back. |
|  | Attitudes towards exercise | N12 (female, 85 years of age): I consider that exercise helps treat diabetes, and I believe it. |
|  |  | N11(female, age 76): Em……In fact, I don’t trust what my friends told me, like what I should eat for my diabetes, I only believe suggestions from healcare professionals in the hospitals, like exercise is good for diabetes. |
|  |  | N15(male, 83 years of age): I keep doing exercise for as long as I can. I know it’s really good for my body. |
|  | Practices of exercise | N13 (female, 90 years of age): My body feels weak now, and all I can do is walk around at home following the walls.  N4 (male, 73 years of age): I used to do morning exercise in the park several months ago, but I haven’t been doing any exercise recently. |
|  |  | N8(male, age 60): I don't usually do exercise, I'm too tired, especially my feet feel weak……you know, I'm a bit lazy.  N6 (female, 69 years of age): I don’t do exercise recently, I’m too busy and I have no time.  N13(female, age 90): I’m not able to do much exercise...now, I’m so weak. |
| Motivators | Desire for health | N3(male, age 66)：Exercise is good for diabetes and I do it because I want to recover quickly. |
|  |  | N2(female, age 63)：I do exercise for health, this is the only reason. |
|  |  | N1 (female, 76 years of age): I keep going for walks even though I’m in hospital, because my feet feel weak, painful, and stiff if I don’t take a walk. |
|  |  | N9(female, age 68)：I think that the less I move my legs, the worse my body gets. So I have to move. |
|  |  | N14 (female, 71 years of age): Yes, the more you lead a sedentary lifestyle, the more trouble your body will run into. |
|  | Positive feelings regarding exercise | N14 (female, 71 years of age): Doing exercise regularly makes me feel more energetic and increases my ability to take care of myself. |
|  |  | N15(male, age 83): This disease makes me feel a little bit tired, but my body feel……much easier and won't be so……numb and……weak like before if I keep walking.  N9(female, age 68): I feel very relaxed...and easy after doing exercise every time, and my lower back won’t be painful, it’s really good! |
|  | Social support | N3 (male, 66 years of age): I’m quite willing to do exercise when my children support and encourage me, because they give me energy and courage. |
|  |  | N11 (female, 76 years of age): It’s really interesting to do exercise with friends. I can chat with them during exercise, so I don’t even feel tired and the time passes quickly. I quite enjoy it.  N1 (female, 76 years of age): I won’t go for a walk unless my best friends call me to, I feel boring if I do exercise alone.  N5 (female, 77 years of age): My children always said‘Mom, you’d better walk more, I’ll go with if you like.’ |
| Barriers | Physical discomfort | N8 (male, 60 years of age): My feet feel very weak and my lower back feels painful because of my lumbar intervertebral disc, so it’s difficult for me to stand up, let alone exercise. |
|  |  | N5(female, age 77)：My feet feel painful and weak so I can’t move quite often, and I get tired easily, which makes me fall easily, it’s horrible.  N6 (female, 69 years of age): My lower back feels heavily pain, so I like lying on bed, I don’t like to do exercise.  N9 (female, 68 years of age): I got stroke before, it’s difficult to move my body. |
|  | Psychological factors | N5 (female, 77 years of age): Sometimes I feel unhappy and I just want to lie in bed alone, which means that I don’t have the energy or enthusiasm to do exercise. |
|  |  | N1(female, age 76): I don’t wanna do anything when I’ m in a bad mood, especially when I’m sad……you know.  N6 (female, 69 years of age): I don’t like to do square dance, I always feel that everybody is watching me and laughing at me, it makes me feel unhappy and upset, so I don’t do exercise. |
|  | Poor exercise conditions (weather, exercise area, assistive devices, time) | N15 (male, 83 years of age): I insist on going for a walk every day in good weather, but if it’s raining I don’t go out because……I’m too old and I am…afraid of falling, which would be terrible and dangerous.  N2 (female, 63 years of age): I feel very busy and tired with all the grocery shopping, cooking, laundry, and looking after my grandchildren, and I have absolutely no time or energy to exercise. |
|  |  | N13(female, age 90)：There are no areas for me to do exercise now, no, no avaiable places for me like before. And I’m so weak that I need a walking frame to help me.  N7(male, age 62): The park I do exercise is too far from my home, and recently I have to take care of my grandchildren, so it’s inconvenient. |
| Attitudes towards professional exercise instruction | Urgent need for exercise instruction | N9 (female, 68 years of age): I definitely need healthcare professionals’ guidance about exercise, I really need that, but every time I feel like the doctors and nurses just tell me to exercise more, which is too vague. |
|  |  | N14(female, age71)：I would like to accept exercise instrutions from you, yes, since I want to be healthier and it will ease the burden on my children. And I’m willing to follow doctors and nurses’ advice, I wouldn’t have come to the hospital if I don’t trust you professionals.  N3 (male, 66 years of age): It would be better if the healthcare professionals come to help me. Yes, I need that.  N15 (male, 83 years of age): That (exercise instruction) is good! Who would refuse?  N7(male, age 62): I’d like to! I’d appreciate it if you teach me how to do exercise. |
|  | Fear of intensity of the instructed exercise | N12 (female, 85 years of age): I wouldn’t accept professional guidance unless I’m able to do it. I wonder whether I could do it or not, because I would feel frustrated and useless if I couldn’t achieve the exercise goals. |
|  |  | N11(female, age 76)：One of the most important things is that I wonder whether I am able to do it or not, I’m quite worried about the intensity of the exercise instructions and my body as well.  N15 (male, 83 years of age): But I’m afraid of the intensity of your professional exercise guidance, what if I couldn’t achieve that? |
|  | Financial constraints | N10 (male, 68 years of age): It would be better if the exercise instruction was free. That would be the most helpful and important thing for me. We don’t have much money and my medicine is expensive, so life is really hard. We are afraid to get sick because it’s really expensive. |
|  |  | N2(female, age 63)：I’m williing to get professional exercise suggestions from healthcare professionals if it’s free, why not?  N3 (male, 66 years of age): Oh, is that free? I’m afraid that I can’t afford it, because my medicine fee is quite high, I don’t have extra money. |
